# Clinical Outcomes in Patients With Quadricuspid vs Bicuspid Aortic Valve

**DOI:** 10.1001/jamanetworkopen.2025.24915

**Published:** 2025-08-04

**Authors:** Jingnan Zhang, Fang Fang, Zhiyuan Xia, Wenhao Zhu, Christopher Tze-Wei Tsang, Qingwen Ren, Jiayi Huang, Ran Guo, Wenli Gu, Haochen Xuan, Yap-Hang Chan, Tai-Leung Chan, Alan C. Yeung, Victoria Delgado, Xiangbin Pan, Gejun Zhang, Kai-Hang Yiu

**Affiliations:** 1 Division of Cardiology, Department of Medicine, The University of Hong Kong–Shenzhen Hospital, Shenzhen, China; 2 Division of Cardiology, Department of Medicine, The University of Hong Kong, Queen Mary Hospital, Hong Kong SAR, China; 3 Structural Heart Disease Center, Fuwai Hospital, National Center for Cardiovascular Disease, Beijing, China; 4 Cardiothoracic Surgery Unit, The University of Hong Kong, Queen Mary Hospital, Hong Kong SAR, China; 5 Division of Cardiovascular Medicine, Stanford University Medical Center, Stanford, California; 6 University Hospital, Germans Trias i Pujol Hospital, Badalona, Spain

## Abstract

**Importance:**

Congenital aortic valve disease predominantly manifests as bicuspid aortic valve (BAV), while quadricuspid aortic valve (QAV) is considerably rarer. Whether outcomes vary between these 2 types of congenital aortic valve disease remains unknown.

**Objective:**

To compare outcomes between patients with QAV and patients with BAV.

**Design, Setting, and Participants:**

In this cohort study, patients diagnosed with QAV or BAV were retrospectively identified from echocardiographic examinations performed from January 1, 2011, to December 31, 2023, at 2 tertiary hospitals in China. Patients with QAV were propensity score matched 1:5 by age and sex with patients with BAV.

**Exposure:**

Presence of QAV or BAV.

**Main Outcomes and Measures:**

Outcomes of interest included aortic valve intervention (surgical or transcatheter), aortic surgery, aortic dissection, infective endocarditis (IE), heart failure hospitalization (HFH), and all-cause death. Morbidity rates were estimated using the cumulative incidence function to account for competing risk of death. Relative survival was calculated as the ratio of observed survival to the expected survival in the general population.

**Results:**

From 2 945 132 echocardiographic examinations, 139 patients with QAV (median age, 54.8 [IQR, 46.2-63.9] years; 85 [61.2%] male) and 695 with BAV (median age, 55.4 [IQR, 45.5-64.2] years; 425 [61.2%] male) were included. Over a median follow-up of 4.8 years (IQR, 2.4-8.0 years), patients with QAV had a similar incidence of aortic valve intervention (77.4% [95% CI, 64.5%-86.1%] vs 75.7% [95% CI, 71.1%-79.8%]; *P* = .26) but lower incidence of aortic surgery (5.1% [95% CI, 0.9%-15.0%] vs 33.8% [95% CI, 28.4%-39.2%]; *P* < .001) and IE (not observed vs 9.6% [95% CI, 6.4%-13.5%]; *P* = .01) compared with patients with BAV. Aortic dissection was rare in both groups (QAV, 0.9% [95% CI, 0.1%-4.1%]; BAV, 3.6% [95% CI, 1.8%-6.4%]; *P* = .57). Compared with BAV, QAV morphology was associated with an increased risk of HFH after multivariable adjustments (adjusted hazard ratio [AHR], 2.52; 95% CI, 1.51-4.20; *P* < .001). Presence of cardiomyopathies was an independent factor associated with HFH in patients with QAV (AHR, 4.27; 95% CI, 2.26-8.07; *P* < .001). The 5-year relative survival rate compared with the age- and sex-matched general population was 102.9% (95% CI, 97.9%-107.2%) for patients with QAV and 102.4% (95% CI, 99.3%-104.8%) for those with BAV.

**Conclusions and Relevance:**

In this large, retrospective cohort study, both QAV and BAV were associated with a high risk of aortic valve intervention. Individuals with QAV had a greater risk of HFH, while those with BAV had higher incidence of IE and aortic surgery. Despite the substantial morbidity burden, survival in both groups was similar to that in the general population.

## Key Points

**Question:**

Do morbidity and survival outcomes differ between patients with quadricuspid aortic valve (QAV) vs bicuspid aortic valve (BAV)?

**Findings:**

In this cohort study of 139 patients with QAV and 695 with BAV matched 1:5 by age and sex, those with QAV had a similarly high incidence of aortic valve intervention, more coexisting cardiomyopathies, and an increased risk of heart failure hospitalization compared with those with BAV. Both groups had survival comparable to that of the age- and sex-matched general population.

**Meaning:**

Patients with QAV may benefit from closer surveillance of valve function and systematic screening for cardiomyopathy.

## Teaser

This cohort study compares morbidity and survival outcomes among individuals with quadricuspid vs bicuspid congenital aortic valve disease.

## Introduction

Congenital malformation of the aortic valve frequently presents as a bicuspid aortic valve (BAV), whereas a quadricuspid aortic valve (QAV) is considerably less common.^1^ The estimated prevalence of BAV in the general population ranges from 0.8% to 1%,^2^ representing the most prevalent congenital heart defect.^3^ Conversely, the prevalence of QAV remains unreported in population-based studies, but retrospective investigations based on tertiary referral hospitals suggest an incidence of less than 0.01%.^1,4,5^ Given its rarity, clinical evaluation strategies for QAV primarily draw on evidence from BAV, thus raising questions about the effectiveness of management. To address this issue, it is essential to understand the similarities and differences in outcomes of these 2 types of congenital aortic valve disease.

Extensive studies have revealed that patients with BAV have a substantial morbidity burden, including various types of valvular dysfunction, aortopathy, heart failure, and endocarditis, but contemporary management leads to favorable survival outcomes.^6,7^ Regarding patients with QAV, current knowledge suggests that they are at risk of aortic regurgitation and may require aortic valve replacement. Similar to BAV, QAV can coexist with other cardiac conditions, such as aortic dilation, coronary anomalies, and inherited cardiomyopathies.^8,9,10^ However, most of these findings of QAV were incidental, and to our knowledge, no extensive study has yet consolidated them using multiple imaging modalities and longer-term follow-up data. Based on a large patient cohort, the current study aimed to (1) evaluate valvular function, aortic dimensions, valve calcification, and concomitant cardiac anomalies in patients with QAV and BAV; (2) compare the lifetime morbidity burden among patients with QAV and BAV; and (3) assess relative survival in the study groups compared with that in the general population.

## Methods

### Patient Population

In this cohort study, between January 1, 2011, and December 31, 2023, patients diagnosed with QAV or BAV were identified from echocardiographic examinations performed at 2 tertiary hospitals in China (Fuwai Hospital and Queen Mary Hospital). Cases were identified by searching the echocardiographic database for diagnostic keywords including *quadricuspid aortic valve* and *bicuspid aortic valve*. Patients with any of the following conditions were excluded: indeterminate aortic valve morphology, complex congenital heart defects,^3^ prior aortic valve interventions, prior aortic surgery, missing echocardiographic images, or poor imaging quality. For patients with multiple examinations, the first echocardiogram was deemed as the baseline examination. Consequently, consecutive patients with QAV were included and further matched 1:5 by age and sex with patients with BAV using propensity scores. The rationale for the matching method is provided in the eMethods in Supplement 1. Baseline comorbidities, echocardiographic parameters, multimodality imaging data, and follow-up information were collected for this matched cohort (eFigure 1 in Supplement 1). The study protocol was approved by the ethics committees of Fuwai Hospital and Queen Mary Hospital. Patient informed consent was waived due to the retrospective design of the study. We followed the Strengthening the Reporting of Observational Studies in Epidemiology (STROBE) reporting guideline.

### Echocardiography

Transthoracic echocardiography (TTE) was performed using commercially available ultrasonography machines. De novo review and reassessment of echocardiographic images (baseline TTE examination) were conducted by experienced sonographers (J.Z., F.F.). Valve morphology was determined in parasternal short-axis views at aortic valve level (eFigure 2 in Supplement 1). Aortic stenosis and aortic regurgitation were graded as none (none or trivial for aortic regurgitation), mild, moderate, or severe using multiparametric approaches as recommended.^11,12^ Interobserver and intraobserver reproducibility of aortic stenosis and aortic regurgitation grading parameters were demonstrated in a random sample of 20 patients (6 with QAV, 14 with BAV) (eTable 1 in Supplement 1). Patients with less than moderate aortic stenosis and regurgitation were defined as having no hemodynamically significant valvular dysfunction. Significant valvular dysfunction was further categorized as isolated aortic stenosis (moderate to severe aortic stenosis without moderate to severe aortic regurgitation), isolated aortic regurgitation (moderate to severe aortic regurgitation without moderate to severe aortic stenosis), and mixed aortic valve disease (MAVD; concomitant moderate to severe aortic stenosis and moderate to severe aortic regurgitation). Left ventricular ejection fraction (LVEF) was measured by the Simpson biplane method.^13^ Aortic dimensions were measured in the parasternal long-axis view in end diastole at 3 levels: sinus of Valsalva, sinotubular junction, and ascending aorta.

### Multimodality Imaging

Two subsets of patients who received either cardiac computed tomography (CT) or cardiac magnetic resonance imaging (MRI) within 12 months from baseline TTE were reviewed. Scans performed after aortic valve intervention or aortic surgery were disregarded. Referrals for cardiac CT and cardiac MRI in both centers adhered to the class I guideline recommendations.^14,15^ Specifically, in the context of congenital aortic valve disease, cardiac CT was generally indicated for patients with poor echocardiographic imaging quality, those with concomitant cardiac anomalies, those undergoing cardiac surgery or interventions, or those exhibiting symptoms suggestive of coronary artery disease.^15^ Cardiac MRI was typically indicated for patients with poor echocardiographic imaging quality, those with concomitant cardiac anomalies, and all patients suspected of having cardiomyopathy.^14^ Aortic valve calcium score was assessed using the Agatston method on non–contrast-enhanced electrocardiography-gated cardiac CT scans.^16^ Congenital coronary anomalies were identified through cardiac CT angiography for accurate localization of coronary origins and anatomy.^17^ The diagnosis and classification of cardiomyopathy were determined by both echocardiographic imaging and cardiac MRI, in accordance with current guidelines.^18^

### Outcomes

Follow-up information was obtained through medical records (and through links to other local institutions) and telephone or online interviews with the patients and their relatives. In both centers, patients with congenital aortic valve disease are followed up annually (normally twice a year) by clinical consultations and echocardiography. The morbidity end points included aortic valve intervention (either surgical or transcatheter), aortic surgery, aortic dissection, infective endocarditis, and heart failure hospitalization (HFH). The mortality end point was all-cause death. The occurrence of aortic dissection was confirmed by operation records or radiologic imaging. Infective endocarditis was defined as any infection involving native aortic valve that meets the modified Duke Criteria.^19^ Heart failure hospitalization was defined as the occurrence of an inpatient or outpatient hospital visit due to heart failure conditions (eTable 3 in Supplement 1). To characterize the lifetime morbidity burden, the date of birth was considered as the starting point for event-free survival analyses, with age being the timescale. When analyzing HFH, we also used the diagnosis date (baseline TTE) as time 0 because HFH events before the establishment of patient reporting systems (since the 1990s) might not be fully documented. The analysis of mortality starting at the diagnosis date allowed a comparison of the observed mortality in study groups with that of the age- and sex-matched general population.

### Statistical Analysis

Continuous variables were presented as means with SDs or medians with IQRs, while categorical variables were presented as numbers and percentages. A *t* test was used to compare continuous variables, while a Pearson χ^2^ test was used for categorical variables. Proportions of missing data are provided in eFigure 3 in Supplement 1. The Kaplan-Meier method was used to calculate mortality in study groups, while the cumulative incidence function was applied to estimate cumulative morbidity rates to account for the competing risk of death. For the analysis of HFH starting from the date of diagnosis, several methodologic approaches were implemented to mitigate potential time bias related to age at diagnosis. First, patients with QAV and BAV were matched by age at diagnosis (per our study design) and further stratified by valvular function for comparative analysis. Second, patients with preexisting or baseline heart failure were excluded from this analysis. Third, multivariable Fine-Gray regression models were used to evaluate the independent association of valve morphology with HFH. We further developed 2 Fine-Gray regression models to separately identify factors associated with incident HFH in the 2 study groups. The assumption of the Fine-Gray model was checked based on cumulative sums of residuals. To compare the mortality with that in the general population, each patient with QAV or BAV was matched to the expected annual survival rate of their corresponding general population based on age, sex, and calendar year under observation. All control data were derived from the life tables in government census records in China (National Bureau of Statistics of China^20^). Relative survival rates were computed as the ratio of observed survival in the study groups to expected survival in the general population. Statistical significance was defined as 2-tailed *P* < .05. Data analysis was performed using R, version 4.5.0 (R Project for Statistical Computing).

## Results

### Baseline and Echocardiographic Findings

From a total of 2 945 132 echocardiographic examinations, this comprehensive comparison included 139 patients with QAV (median age, 54.8 years [IQR, 46.2-63.9 years]; 54 [38.8%] female; 85 [61.2%] male) and 695 with BAV (median age, 55.4 years [IQR, 45.5-64.2 years]; 270 [38.8%] female, 425 [61.2%] male). Baseline characteristics are provided in Table 1. Follow-up information was obtained from 137 patients with QAV (98.6%) and 682 with BAV (98.1%) (eTable 2 in Supplement 1). Among the patients, 35 with QAV (25.2%) and 245 with BAV (35.3%) did not show hemodynamically significant valvular dysfunction (*P* < .001). Patients with QAV predominantly had isolated aortic regurgitation (99 [71.2%]), while patients with BAV had various valvular dysfunctions. Patients with QAV had a lower LVEF and larger LV sizes and volumes but lower LV wall thickness than did those with BAV. Aortic measurements revealed that patients with QAV had a smaller ascending aorta diameter (median, 3.5 cm [IQR, 3.2-4.0 cm] vs 3.9 cm [IQR, 3.3-4.5 cm]; *P* < .001) and a lower proportion with aortic dilation greater than 4 cm (43 [30.9%] vs 321 [46.2%]; *P* = .001).

**Table 1.** Baseline Characteristics of the Overall Cohort

Characteristic	Patients^a^		*P* value
	QAV (n = 139)	BAV (n = 695)	
**Demographics**			
Age, median (IQR), y	54.8 (46.2-63.9)	55.4 (45.5-64.2)	.90
Sex			
Female	54 (38.8)	270 (38.8)	>.99
Male	85 (61.2)	425 (61.2)	
Height, median (IQR), cm	168.0 (160.0-172.0)	165.0 (158.0-172.0)	.001
Weight, median (IQR), kg	68.0 (60.0-77.0)	65.6 (56.4-75.0)	.02
BSA, median (IQR), m^2^	1.8 (1.6-1.9)	1.7 (1.6-1.9)	.005
BMI, median (IQR)	24.6 (22.2-26.5)	24.0 (21.8-26.6)	.42
**Comorbidities**			
Hypertension	74 (53.2)	242 (34.8)	<.001
Hyperlipidemia	54 (38.8)	143 (20.6)	<.001
Diabetes	14 (10.1)	87 (12.5)	.50
Coronary artery disease	23 (16.5)	152 (21.9)	.19
Atrial fibrillation	8 (5.8)	27 (3.9)	.44
Prior or baseline heart failure	17 (12.2)	70 (10.1)	.09
**Echocardiography**			
Valvular function			
No hemodynamically significant valvular dysfunction	35 (25.2)	245 (35.3)	<.001
Isolated aortic stenosis	1 (0.7)	261 (37.6)	
Isolated aortic regurgitation	99 (71.2)	133 (19.1)	
MAVD	4 (2.9)	56 (8.1)	
Aortic stenosis			
None	120 (86.3)	266 (38.3)	<.001
Mild	14 (10.1)	112 (16.1)	
Moderate	5 (3.6)	101 (14.5)	
Severe	0	216 (31.1)	
Aortic regurgitation			
None or trivial	13 (9.4)	392 (56.4)	<.001
Mild	22 (15.8)	114 (16.4)	
Moderate	65 (46.8)	87 (12.5)	
Severe	39 (28.1)	102 (14.7)	
Aortic valve assessment, median (IQR)			
Peak gradient, mm Hg	13.0 (9.0-21.2)	36.0 (17.0-70.6)	<.001
Mean gradient, mm Hg	6.5 (4.5-10.6)	20.0 (9.0-42.0)	<.001
Peak velocity, m/s	1.8 (1.5-2.4)	3.1 (2.0-4.3)	<.001
LVEF, mean (SD), %	56.7 (10.8)	59.4 (11.1)	.008
LVESD, median (IQR), cm	3.9 (3.2-5.2)	3.1 (2.7-3.7)	<.001
LVEDD, median (IQR), cm	5.8 (5.4-6.6)	4.7 (4.2-5.4)	<.001
IVS, median (IQR), cm	1.0 (0.9-1.1)	1.4 (1.1-1.7)	<.001
PWT, median (IQR), cm	0.9 (0.8-1.1)	1.1 (0.9-1.3)	<.001
LVESV, median (IQR), mL	62.0 (44.0-99.8)	38.0 (25.0-58.0)	<.001
LVEDV, median (IQR), mL	160.0 (141.0-235.0)	100.0 (74.0-134.5)	<.001
Aortic dimensions, median (IQR), cm			
Ascending aorta diameter	3.5 (3.2-4.0)	3.9 (3.3-4.5)	<.001
Sinus of Valsalva diameter	3.3 (3.2-3.6)	3.3 (3.0-3.8)	.86
Sinotubular junction diameter	2.8 (2.8-2.8)	3.0 (2.6-3.4)	.37
Aorta diameter >4 cm	43 (30.9)	321 (46.2)	.001

Abbreviations: BAV, bicuspid aortic valve; BMI, body mass index (calculated as weight in kilograms divided by height in meters squared); BSA, body surface area; IVS, interventricular septal diameter; LVEDD, left ventricular end-diastolic diameter; LVEDV, left ventricular end-diastolic volume; LVEF, left ventricular ejection fraction; LVESD, left ventricular end-systolic diameter; LVESV, left ventricular end-systolic volume; MAVD, mixed aortic valve disease; PWT, posterior wall thickness; QAV, quadricuspid aortic valve.

^a^ Data are presented as number (percentage) of patients unless otherwise indicated.

Among patients without hemodynamically significant valvular dysfunction (eTable 4 in Supplement 1), those with QAV continued to have a lower LVEF than patients with BAV (mean [SD], 57.2% [10.0%] vs 61.6% [7.5%]; *P* = .002) despite comparable demographics and comorbidities. Although it was without hemodynamically significant valvular dysfunction, aortic dilation (aortic diameter >4 cm) was still observed in 4 of 35 patients with QAV (11.4%) and 85 of 245 with BAV (34.7%) (*P* = .01). Comparison of patients with valvular dysfunction is provided in eTable 5 in Supplement 1.

### Findings From Multimodality Imaging

A total of 107 patients with QAV (77.0%) and 250 with BAV (36.0%) underwent cardiac CT scanning. Baseline characteristics are provided in eTable 6 in Supplement 1. The aortic valve calcium score was significantly lower in patients with QAV compared with those with BAV (mean (SD), 17.4 [90.9] Agatston units [AU] vs 714.5 [927.4] AU; *P* < .001). The high aortic valve calcium score in patients with BAV was mainly attributable to the groups with isolated aortic stenosis and MAVD (eFigure 4 in Supplement 1). Coronary artery anomalies were found in 5 QAV cases (4.7%) and 14 BAV cases (5.6%) (*P* = .87) (eFigure 5 in Supplement 1).

**Figure 1.**
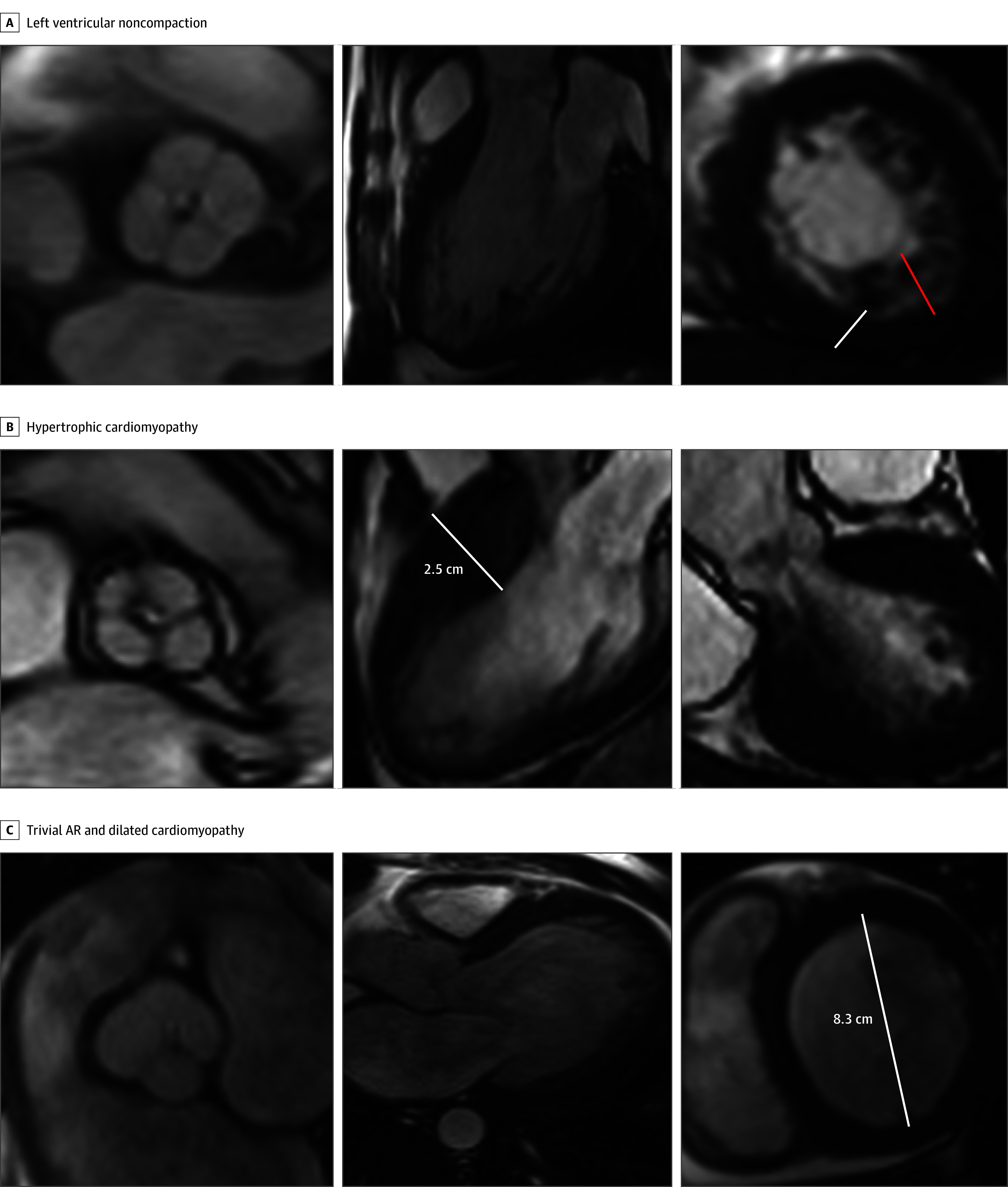
Coexistence of Quadricuspid Aortic Valve (QAV) and Cardiomyopathy Detected From Cardiac Magnetic Resonance Imaging A, Patient with QAV diagnosed with left ventricular (LV) noncompaction with a noncompacted (red line) to compacted (white line) ratio greater than 2. B, Patient with QAV diagnosed with hypertrophic cardiomyopathy with interventricular septal thickness of 2.5 cm (white line). C, Patient with QAV diagnosed with trivial aortic regurgitation (AR) and dilated cardiomyopathy with cavity size of 8.3 cm (white line). Images on the left depict the cross-sectional aortic valve view and the middle images, the LV 3-chamber view. Images on the right in A and C display the true LV short-axis view and in B, a coronal view of the LV outflow tract.

Cardiac MRI was performed for 21 of all patients with QAV (15.1%) and 35 with BAV (5.0%). Baseline characteristics of patients referred to cardiac MRI are provided in eTable 7 in Supplement 1. Through a comprehensive imaging evaluation, cardiomyopathy was identified in 8 patients with QAV (5.8%), of whom 3 (37.5%) had hypertrophic cardiomyopathy, 2 (25.0%) had dilated cardiomyopathy, and 3 (37.5%) had LV noncompaction. Clinical features and imaging findings of patients with coexisting QAV and cardiomyopathy are provided in Figure 1 and eTable 8 in Supplement 1. In contrast, a lower prevalence of cardiomyopathy was observed in patients with BAV (5 [0.1%]), of whom 3 (60.0%) had hypertrophic cardiomyopathy, 1 (20.0%) had dilated cardiomyopathy, and 1 (20.0%) had LV noncompaction.

### Lifetime Morbidity Burden

**Figure 2.**
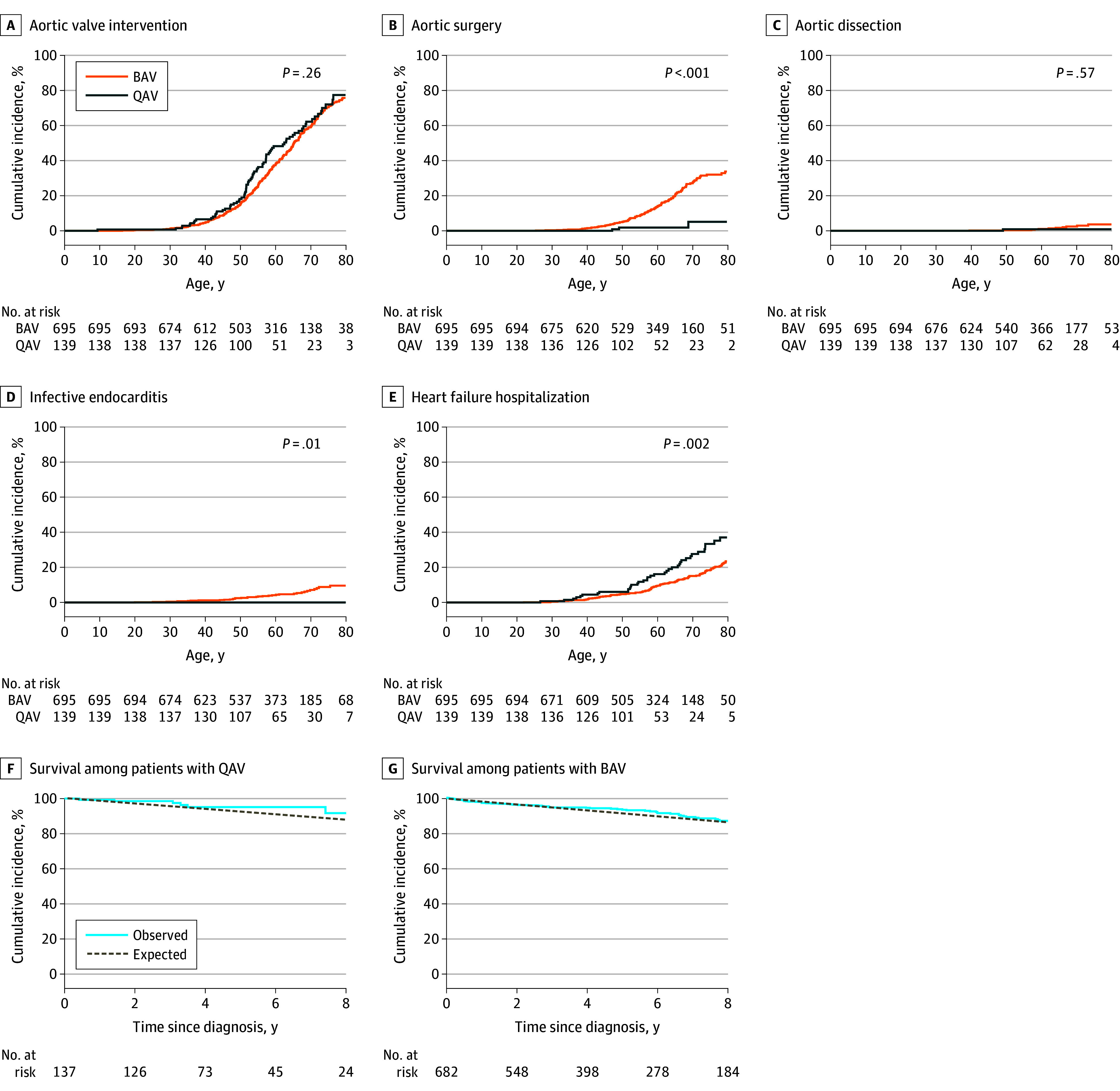
Outcomes in Patients With Quadricuspid Aortic Valve (QAV) and Bicuspid Aortic Valve (BAV) A-E, Date of birth was used as time 0 to assess the lifetime incidence of morbidity end points by age. F and G, Mortality analysis starting from the diagnosis date allowed a comparison with the expected survival in the age- and sex-matched general population.

The median follow-up period of the overall cohort was 4.8 years (IQR, 2.4-8.0 years), with no significant difference between patients with QAV (4.2 years [IQR, 2.8-7.8 years]) and those with BAV (4.7 years [IQR, 2.3-8.6 years]) (*P* = .29). Clinical characteristics and outcomes in the group with BAV vs the group with QAV are shown in Table 2. From birth to age 80 years, the cumulative incidence of aortic valve intervention was comparably high in both groups (77.4% [95% CI, 64.5%-86.1%] for QAV vs 75.7% [95% CI, 71.1%-79.8%] for BAV; *P* = .26) (Table 2 and Figure 2A). In contrast, the incidence of aortic surgery was significantly lower in patients with QAV compared with those with BAV (5.1% [95% CI, 0.9%-15.0%] vs 33.8% [95% CI, 28.4%-39.2%]; *P* < .001) (Figure 2B). Aortic dissection occurred in 1 patient with QAV (0.8%) and 17 patients with BAV (2.4%), resulting in a cumulative incidence of 0.9% (95% CI, 0.1%-4.1%) for QAV and 3.6% (95% CI, 1.8%-6.4%) for BAV by age 80 years (*P* = .57) (Figure 2C). The single QAV case of aortic dissection was classified as type B. Among the 17 patients with BAV and aortic dissection, 15 cases (88.2%) were classified as type A and 2 (11.8%) as type B. Infective endocarditis was not observed in patients with QAV; however, patients with BAV had a lifetime incidence of 9.6% (95% CI, 6.4%-13.5%) by age 80 years (*P* = .01) (Figure 2D). HFH events occurred in 38 patients with QAV (27.3%) and 121 patients with BAV (17.4%). By age 80 years, the cumulative incidence of HFH was significantly higher in patients with QAV (37.0% [95% CI, 27.0%-47.0%]) compared with patients with BAV (23.5% [95% CI, 19.4%-27.7%]) (*P* = .002) (Figure 2E).

**Table 2.** Outcomes in Patients With QAV and BAV

Outcome	Commonness^a^	
	QAV	BAV
Aortic valve pathology		
Aortic stenosis	Rare	Substantial
Aortic regurgitation	Substantial	Common
MAVD	Rare	Uncommon
Aortic valve intervention, cumulative incidence (95% CI), %	77.4 (64.5-86.1)	75.7 (71.1-79.8)
Aortopathy		
Aortic dilation	Common	Common
Aortic surgery, cumulative incidence, % (95% CI)	5.1 (0.9-15.0)	33.8 (28.4-39.2)
Aortic dissection, cumulative incidence, % (95% CI)	0.9 (0.1-4.1)	3.6 (1.8-6.4)
Other		
Cardiomyopathy	Uncommon	Rare
Infective endocarditis, cumulative incidence, % (95% CI)	Not observed	9.6 (6.4-13.5)
Heart failure hospitalization, cumulative incidence, % (95% CI)	37.0 (27.0-47.0)	23.5 (19.4-27.7)
5-y Relative survival, % (95% CI)^b^	102.9 (97.9-107.2)	102.4 (99.3-104.8)

Abbreviations: BAV, bicuspid aortic valve; MAVD, mixed aortic valve disease; QAV, quadricuspid aortic valve.

^a^ Baseline prevalences are categorized as substantial, common, uncommon, and rare, corresponding to prevalence rates of over 50%, 30% to 50%, 5% to 30%, and less than 5%, respectively. All morbidity rates are reported as cumulative incidences. Interpretations should be made with caution due to the limited sample size.

^b^ In both groups, survival was comparable to that in the general population.

### HFH Risk in Patients With QAV

For HFH analysis starting from the diagnosis date, 87 patients with prior or baseline heart failure (17 with QAV [12.2%] and 70 with BAV [10.1%]) were excluded. QAV morphology was independently associated with a higher risk of HFH compared with BAV morphology after adjusting for baseline confounders (adjusted hazard ratio [AHR], 2.52; 95% CI, 1.51-4.20; *P* < .001) (Table 3). This increased risk persisted among patients without hemodynamically significant valvular dysfunction (AHR, 5.04; 95% CI, 2.34-10.80; *P* < .001). Factors including male sex (AHR, 2.37; 95% CI, 1.08-5.20; *P* = .03), atrial fibrillation (AHR, 3.05; 95% CI, 1.08-8.61; *P* = .03), the presence of cardiomyopathies (AHR, 4.27; 95% CI, 2.26-8.07; *P* < .001), and severe aortic regurgitation (AHR, 2.63; 95% CI, 1.04-6.64; *P* = .04) were independently associated with HFH in the group with QAV. In patients with BAV, the factors associated with HFH were age (AHR, 1.02; 95% CI, 1.01-1.04; *P* = .003), male sex (AHR, 1.99; 95% CI, 1.34-2.96; *P* < .001), diabetes (AHR, 1.77; 95% CI, 1.07-2.93; *P* = .02), atrial fibrillation (AHR, 2.56; 95% CI, 1.29-5.08; *P* = .007), severe aortic stenosis (AHR, 2.26; 95% CI, 1.33-3.85; *P* = .003), and severe aortic regurgitation (AHR, 3.12; 95% CI, 1.94-5.01; *P* < .001) (eTable 9 in Supplement 1).

**Table 3.** Association Between Valve Morphology and Risk of HFH^**a**^

Characteristic	Patients								
	Overall cohort			Valvular dysfunction					
				Not hemodynamically significant			Hemodynamically significant		
	BAV	QAV	*P* value	BAV	QAV	*P* value	BAV	QAV	*P* value
Events/person-years	55/2293.6	23/293.6	NA	11/1247.3	6/147.4	NA	44/1046.2	17/147.4	NA
Incidence rate, per 100 person-years (95% CI)	2.49 (1.90-3.27)	8.17 (5.41-12.07)	NA	0.88 (0.46-1.62)	4.07 (1.66-9.04)	NA	4.21 (2.60-4.74)	11.5 (7.06-18.09)	NA
HR (95% CI)	1 [Reference]	2.34 (1.45-3.77)	<.001	1 [Reference]	4.30 (2.15-8.64)	<.001	1 [Reference]	1.68 (0.96-2.94)	.06
Adjusted HR (95% CI)^b^	1 [Reference]	2.52 (1.51-4.20)	<.001	1 [Reference]	5.04 (2.34-10.80)	<.001	1 [Reference]	1.75 (0.95-3.32)	.07

Abbreviations: BAV, bicuspid aortic valve; HFH, heart failure hospitalization; HR, hazard ratio; NA, not applicable; QAV, quadricuspid aortic valve.

^a^ Patients with prior or baseline heart failure were excluded from this analysis.

^b^ Multivariable Fine-Gray HR model with all-cause mortality as the competing risk, adjusting for age, male sex, hypertension, hyperlipidemia, diabetes, coronary artery disease, atrial fibrillation, and aortic valve intervention (as time-dependent covariate).

### Survival

All-cause death was documented in 7 patients with QAV (5.0%) and 68 patients with BAV (9.8%). On Kaplan-Meier analysis, the observed 5-year survival was 95.2% (95% CI, 91.0%-99.5%) for patients with QAV and 93.1% (95% CI, 91.0%-95.3%) for those with BAV (Figure 2F and G). Compared with the age- and sex-matched general population, the 5-year relative survival rates were 102.9% (95% CI, 97.9%-107.2%) for patients with QAV and 102.4% (95% CI, 99.3%-104.8%) for those with BAV.

## Discussion

In, to our knowledge, the largest series of patients with QAV studied to date, we found similarities and differences in their clinical characteristics and outcomes compared with patients with BAV. The first principal finding was that patients with QAV mainly had isolated aortic regurgitation, while those with BAV had various types of valvular dysfunction and more pronounced aortic dilation. Second, multimodality imaging data revealed a lower level of valvular calcification and a higher prevalence of coexisting cardiomyopathies in patients with QAV than in those with BAV. Third, during the follow-up period, aortic valve intervention was frequently observed in both groups. While QAV was associated with lower incidence of aortic complications and infective endocarditis, it was associated with an elevated risk of HFH. Notably, the presence of cardiomyopathies emerged as an independent factor associated with HFH in patients with QAV. Fourth, the survival rates in both groups were comparable with that in the age- and sex-matched general population.

The primary contributor to lifetime morbidity in individuals with QAV and BAV is progressive valve dysfunction.^6^ Our study made a novel contribution by documenting the parallel age-dependent progression of valve dysfunction in both patients with QAV and patients with BAV, for whom the need for aortic valve intervention markedly escalated after age 40 years. The generalizability of the results is reinforced by consistent outcomes for patients with BAV reported by prior studies.^6,21^ As such, one might cautiously project that individuals diagnosed with QAV may have a high likelihood of requiring aortic valve treatment during their lifetime. Furthermore, we observed significant hemodynamic differences between the 2 groups likely attributable to their distinct anatomic features and variations in valve calcium accumulation. In cases of BAV, aortic regurgitation predominantly affects young males as a result of aortic root dilation or valve prolapse,^22^ while the prevalence of aortic stenosis and MAVD increases with age, exhibiting a robust correlation with valve calcification.^23^ In the context of QAV, retrograde flow arises from asynchronous leaflet coaptation and an unsealed orifice during diastole.^24,25^ Histologic studies have revealed substantial leaflet thickening, distortion, and fibrosis in the accessory leaflet, which might trigger aortic regurgitation progression in patients with QAV.^26,27^ Future research should focus on identifying potential predictors for aortic regurgitation progression to aid in the treatment of patients with QAV.

Our study found that patients with QAV had smaller baseline aortic dimensions than patients with BAV and low incidence of aortic surgery and aortic dissection. Despite fewer patients undergoing prophylactic aortic surgery as compared with BAV, the incidence of aortic dissection in patients with QAV remained significantly low. Thus, we speculate that QAV aortopathy might signify a less aggressive condition with a slower aortic growth rate and fewer aortic complications compared with BAV. This finding may be partially elucidated by a recent 4-dimensional flow cardiac MRI study showing that QAV exhibited a more concentric jet flow toward the aorta and lower blood velocity than BAV,^28^ thereby resulting in reduced wall shear stress and minimal loss of aortic wall elastic fibers.^29^ However, of note, aortic dilation in QAV should not be dismissed, as its prevalence in this study remained significantly higher than general population estimates (1.8% for ascending aortic aneurysm, according to the Copenhagen General Population Study).^30^ Aortic imaging surveillance should also be considered for individuals with QAV, and clinical management strategies for BAV aortopathy could provide valuable insights for the care of these individuals.^31^ While infective endocarditis has been previously reported in patients with QAV,^32,33^ it was not observed in the current study, perhaps indicating a low incidence of this complication. Conversely, infective endocarditis is a more prevalent condition in patients with BAV and carries a high morbidity risk.^34^ This difference is likely explained by the higher-velocity flow and irregular valve anatomy (eg, the presence of raphe, calcification) in BAV, which create turbulence known to be associated with endocarditis.^28,35^ Nonetheless, clinicians should remain vigilant regarding the potential risk of infective endocarditis in patients with QAV until a better understanding of its clinical presentation is gained.

A notable concern arises due to the lower baseline LVEF and increased risk of HFH in patients with QAV as highlighted in our study, a finding that to our knowledge has not been reported previously. Importantly, this heightened risk persisted after excluding patients with valvular dysfunction, suggesting that the underlying myocardial disorder was independent of valve hemodynamics. Using multimodality imaging data, we further found that patients with QAV had a higher prevalence of coexisting cardiomyopathies, which emerged as a significant factor associated with subsequent HFH. This distinctive feature aligns with prior case reports of QAV documenting its coexistence with various inherited cardiomyopathies, encompassing hypertrophic cardiomyopathy, dilated cardiomyopathy, and LV noncompaction.^36,37,38^ Despite the heterogeneity within cardiomyopathy categories, this phenomenon is mechanically plausible, as sequence variations in the same gene can manifest as different myocardial disorders.^39^ Notably, a recent study identified a shared genetic basis for LV noncompaction and BAV involving the MIB1-NOTCH pathway.^40^ Therefore, systematic screening incorporating multimodality imaging and genetic tests is needed to further investigate the accurate prevalence of inherited cardiomyopathy in patients with congenital aortic valve disease. In addition to cardiomyopathies, patients with QAV may experience accumulated LV damage due to the longstanding hemodynamic abnormalities, probably contributing to high HFH burden. A previous study demonstrated that BAV was independently associated with a higher cumulative incidence of postoperative HFH than was tricuspid aortic valve in the context of severe aortic stenosis.^41^ Accordingly, additional research is imperative to ascertain the ideal timing for valve intervention in patients with BAV and QAV to mitigate the risk of HFH.

The ultimate goal of managing care for patients with congenital aortic valve disease is to improve survival. Our study revealed that patients with either type of congenital aortic valve disease had survival comparable to that in the age- and sex-matched general population based on contemporary management strategies in China. The results expanded the current knowledge about QAV by including a different ethnic population, as the only study on QAV was based on patients treated at the Mayo Clinic, where QAV similarly had favorable survival rates, as expected.^4^ However, given the excess mortality associated with HFH,^42^ it is plausible that this observed HFH risk in individuals with QAV might eventually result in survival penalty, which should be further investigated in older cohorts with longer follow-up.

### Limitations

This study has limitations. First, this retrospective, tertiary hospital-based study was open to selection bias by unavoidably missing undiagnosed patients with QAV or BAV in the community or those who died before diagnosis. Moreover, as the diagnosis primarily relied on TTE, some patients with severely calcified valves might have been classified as having indeterminate valve morphology and were not included in the study. Second, we included a matched group with BAV based on age and sex to account for demographic differences between the 2 groups at the time of diagnosis; however, this may not have fully captured the entire spectrum of patients with BAV. Despite this, the sample size for the group with BAV was substantial, and the presentation of valvular dysfunction and morbidity incidence rates was comparable with that in other studies,^6,7,43^ further supporting the representativeness of the cohort with BAV. Third, the multimodality imaging findings might have introduced potential selection bias, as not all patients in the study underwent cardiac CT and cardiac MRI. While the presence of cardiomyopathies was identified as a distinctive feature in QAV, the lack of systematic screening and genetic data may have potentially underestimated QAV prevalence. Fourth, the analyses of HFH and mortality starting from diagnosis date may introduce survival bias in a retrospective setting. Fifth, all outcome analyses in the group with QAV had wide 95% CIs due to the limited sample size, necessitating cautious interpretation of these findings.

## Conclusions

In this large, retrospective cohort study, both QAV and BAV, as typical valvuloaortopathies, were associated with progressive valvular dysfunction and a high incidence of aortic valve intervention. Patients with QAV tended to have smaller aortic dimensions and fewer aortic complications but a significantly higher burden of cardiomyopathies and HFH compared with those with BAV. Despite substantial morbidities, the survival rates for both groups were comparable to that in the general population.

## Notes

Supplement 1.
eMethods
**eTable 1.** Inter- and Intra-Observer Reproducibility of Echocardiographic Measurements in 20 Random Samples **eTable 2.** Numbers of Observed Events in Patients With Quadricuspid and Bicuspid Aortic Valves **eTable 3.** Criteria for Defining Heart Failure Hospitalization in the Study **eTable 4.** Baseline Characteristics of Patients Without Significant Valvular Dysfunction **eTable 5.** Baseline Characteristics in Patients With Valvular Dysfunction **eTable 6.** Baseline Characteristics of Patients Who Underwent Cardiac Computed Tomography Scanning **eTable 7.** Baseline Characteristics of Patients Who Underwent Cardiac Magnetic Resonance Scanning **eTable 8.** Characteristics of Eight Patients With Coexisting Cardiomyopathy and Quadricuspid Aortic Valves **eTable 9.** Factors Associated With Heart Failure Hospitalization in Patients With Quadricuspid and Bicuspid Aortic Valves From Multivariable Regression Analysis **eFigure 1.** Study Flowchart **eFigure 2.** Morphologic Assessment of Aortic Valve Using Echocardiography **eFigure 3.** Proportion of Missing Values for the Main Study Variables **eFigure 4.** Aortic Valve Calcium Score Assessed by Computed Tomography Scanning **eFigure 5.** Coronary Artery Anomalies in Patients With Quadricuspid and Bicuspid Aortic Valves

Supplement 2.
Data Sharing Statement
