# Supplementary material for: Clinical Outcomes in Patients With Quadricuspid vs Bicuspid Aortic Valve
Source: JAMA Netw Open. 2025 Aug 4;8(8):e2524915. doi: 10.1001/jamanetworkopen.2025.24915 (PMC12322796; doi:10.1001/jamanetworkopen.2025.24915)
Supplement: Supplement 1. — eMethods eTable 1. Inter- and Intra-Observer Reproducibility of Echocardiographic Measurements in 20 Random Samples eTable 2. Numbers of Observed Events in Patients With Quadricuspid and Bicuspid Aortic Valves eTable 3. Criteria for Defining Heart Failure Hospitalization in the Study eTable 4. Baseline Characteristics of Patients Without Significant Valvular Dysfunction eTable 5. Baseline Characteristics in Patients With Valvular Dysfunction eTable 6. Baseline Characteristics of Patients Who Underwent Cardiac Computed Tomography Scanning eTable 7. Baseline Characteristics of Patients Who Underwent Cardiac Magnetic Resonance Scanning eTable 8. Characteristics of Eight Patients With Coexisting Cardiomyopathy and Quadricuspid Aortic Valves eTable 9. Factors Associated With Heart Failure Hospitalization in Patients With Quadricuspid and Bicuspid Aortic Valves From Multivariable Regression Analysis eFigure 1. Study Flowchart eFigure 2. Morphologic Assessment of Aortic Valve Using Echocardiography eFigure 3. Proportion of Missing Values for the Main Study Variables eFigure 4. Aortic Valve Calcium Score Assessed by Computed Tomography Scanning eFigure 5. Coronary Artery Anomalies in Patients With Quadricuspid and Bicuspid Aortic Valves [file jamanetwopen-e2524915-s001.pdf]

Supplementary Online Content

Zhang J, Fang F, Xia Z, et al. Clinical outcomes in patients with quadricuspid vs bicuspid aortic valve. *JAMA Netw Open*. 2025;8(8):e2524915. doi:10.1001/jamanetworkopen.2025.24915

eMethods
eTable 1. Inter- and Intra-Observer Reproducibility of Echocardiographic Measurements in 20 Random Samples
eTable 2. Numbers of Observed Events in Patients With Quadricuspid and Bicuspid Aortic Valves
eTable 3. Criteria for Defining Heart Failure Hospitalization in the Study
eTable 4. Baseline Characteristics of Patients Without Significant Valvular Dysfunction
eTable 5. Baseline Characteristics in Patients With Valvular Dysfunction
eTable 6. Baseline Characteristics of Patients Who Underwent Cardiac Computed Tomography Scanning
eTable 7. Baseline Characteristics of Patients Who Underwent Cardiac Magnetic Resonance Scanning
eTable 8. Characteristics of Eight Patients With Coexisting Cardiomyopathy and Quadricuspid Aortic Valves
eTable 9. Factors Associated With Heart Failure Hospitalization in Patients With Quadricuspid and Bicuspid Aortic Valves From Multivariable Regression Analysis
eFigure 1. Study Flowchart
eFigure 2. Morphologic Assessment of Aortic Valve Using Echocardiography
eFigure 3. Proportion of Missing Values for the Main Study Variables
eFigure 4. Aortic Valve Calcium Score Assessed by Computed Tomography Scanning
eFigure 5. Coronary Artery Anomalies in Patients With Quadricuspid and Bicuspid Aortic Valves

This supplementary material has been provided by the authors to give readers additional information about their work.

© 2025 Zhang J et al. *JAMA Network Open*.

eMethods

Matching Methodology

We adopted the age-and-sex propensity score matching method to mitigate demographic differences at diagnosis between patients with quadricuspid aortic valves (QAV) and bicuspid aortic valves (BAV). This method preserved the granularity of age (as continuous variable), a significant determinant of outcomes in patients with BAV.[1] In a simulation study, a 1: 5 propensity matching method was demonstrated to have better performance in the setting of rare disease, in decreasing the bias of estimated risk difference, increasing the balance in covariates, and increasing the coverage of 95% confidence intervals, with a small cost in bias.[3] Furthermore, we estimated the sample size needed to capture outcome differences within a cohort of patients with congenital aortic valve disease. Based on prior studies, we found that the 5-year incidence of surgery on aortic valve or aorta was around 16% for QAV patients and 9% for BAV patients, [3,4] resulting in an assumed relative risk of 1.8. To achieve 80% statistical power and a 2-tailed 5% α level, we calculated that a cohort size of 326 (163 per group) was needed. Consequently, we opted for a 1:5 matching approach, including a total of 834 patients, to balance the bias and ensure sufficient statistical power.

For the propensity score matching, logistic regression was utilized to calculate the propensity score based on age and sex. Nearest neighbor caliper matching was then conducted using the logit of the propensity score, with a caliper set at 0.1 times the standard deviation (SD) of the logit. Data analysis was performed using R version 4.5.0 (R Foundation for Statistical Computing).

Reference:

[1] Avierinos JF, et al. Natural history of asymptomatic patients with normally functioning or minimally dysfunctional bicuspid aortic valve in the community. Circulation. 2008;117(21):2776-2784.

[2] Cenzer I, Boscardin WJ, Berger K. Performance of matching methods in studies of rare diseases: a simulation study. Intractable Rare Dis Res. 2020;9(2):79-88.

© 2025 Zhang J et al. *JAMA Network Open*.

[3] Ammash NM, et al. Quadricuspid Aortic Valve: Characteristics, Associated Structural Cardiovascular Abnormalities, and Clinical Outcomes. Circulation. 2016;133(3):312-319.

[4] Michelena HI, et al. Clinical Outcomes of Adults with Bicuspid Aortic Valve: A European Perspective. Mayo Clin Proc. 2021;96(3):648-657.

© 2025 Zhang J et al. *JAMA Network Open*.

eTable 1. Inter- and intra-observer reproducibility of echocardiographic measurements in 20 random samples [a]

Characteristic	Intra-observer Pearson *r* (95% CI)	*P* value	Inter-observer Pearson *r* (95% CI)	*P* value
AS Grading Parameters				
AV peak velocity	0.96 (0.93–0.98)	<0.001	0.93 (0.90–0.96)	<0.001
AV peak gradient	0.95 (0.92–0.97)	<0.001	0.94 (0.91–0.97)	<0.001
AV mean gradient	0.97 (0.94–0.99)	<0.001	0.91 (0.90–0.95)	<0.001
Aortic valve area	0.96 (0.93–0.98)	<0.001	0.92 (0.89–0.95)	<0.001
AR Grading Parameters				
Vena contracta width [b]	0.93 (0.84–0.97)	< 0.001	0.92 (0.85–0.96)	0.01
Jet width	0.95 (0.91–0.97)	0.002	0.95 (0.90–0.98)	0.003
Jet width/LVOT diameter ratio	0.93 (0.87–0.96)	0.01	0.91 (0.83–0.95)	0.03
AR PHT	0.94 (0.89–0.97)	0.005	0.95 (0.90–0.98)	0.003

[a] The analysis included 6 patients with QAV and 14 with BAV. [b] Vena contracta width was measured as the smallest flow diameter at the level of the aortic valve in the LVOT, normally in the parasternal long-axis views where the regurgitant jet is perpendicular to the direction of ultrasound. AS, aortic stenosis; AV, aortic valve; AR, aortic regurgitation; LVOT, left ventricular outflow tract; PHT, pressure half-time.

© 2025 Zhang J et al. *JAMA Network Open*.

eTable 2. Numbers of observed events in patients with quadricuspid and bicuspid aortic valves [a]

Events [b]	QAV N=137	BAV N=682
Aortic valve intervention	79	388
Aortic surgery	3	133
Aortic dissection	1	17
Infective endocarditis	0	29
Heart failure hospitalization	38	121
All-cause death	7	68

[a] The table only includes patients with follow-up information.

[b] Categories are not mutually exclusive.

© 2025 Zhang J et al. *JAMA Network Open*.

eTable 3. Criteria for defining heart failure hospitalization in the study

Patients met any of the following criteria were considered as having a heart failure hospitalization. The identification of such event was based on administrative codes including *International Classification of Diseases, 9th Revision (ICD-9)* and *Tenth Revision (ICD-10)*, in addition to a comprehensive review of all medical records.
1. The patient was admitted to the hospital with a primary diagnosis of heart failure.
2. The patient exhibited at least one of heart failure symptoms on presentation, together with one physical examination finding and one laboratory finding or invasively measured criterion suggesting new or worsening heart failure condition.
3. The patient received at least one of the following treatments specifically for heart failure: increase in oral diuretic therapy; initiation of intravenous diuretics; initiation of an intravenous vasoactive agent; mechanical circulatory support; or mechanical fluid removal.

© 2025 Zhang J et al. *JAMA Network Open*.

eTable 4. Baseline characteristics of patients without significant valvular dysfunction

Characteristic	QAV N=35	BAV N=245	*P* value
**Demographics**			
Age, median (IQR), years	53.3 [42.3, 62.5]	52.1 [39.8, 61.7]	0.70
Male, No. (%)	20 (57.1)	142 (57.9)	0.92
Height, median (IQR), cm	168.0 [160.0, 178.0]	165.5 [159.4, 173.0]	0.09
Weight, median (IQR), kg	68.0 [64.0, 80.0]	65.0 [58.0, 75.0]	0.11
BSA, median (IQR), m^2	1.8 [1.7, 1.9]	1.8 [1.6, 1.9]	0.07
BMI, median (IQR), kg/m^2	25.3 [22.0, 27.6]	24.1 [21.7, 26.7]	0.43
**Comorbidities**			
Hypertension, No. (%)	17 (48.6)	74 (30.2)	0.05
Hyperlipidemia, No. (%)	10 (28.6)	41 (16.7)	0.14
Diabetes mellitus, No. (%)	2 (5.7)	30 (12.2)	0.39
Coronary artery disease, No. (%)	6 (17.1)	40 (16.3)	1.00
Atrial fibrillation, No. (%)	3 (8.6)	8 (3.3)	0.29
Prior or baseline heart failure, No. (%)	4 (11.4)	12 (4.9)	0.24
**Echocardiography**			
AV peak gradient, median (IQR), mmHg	9.0 [5.8, 15.2]	17.0 [10.0, 23.5]	<0.001
AV mean gradient, median (IQR), mmHg	3.4 [2.2, 5.1]	9.0 [5.0, 13.0]	<0.001
AV peak velocity median (IQR), m/s	1.5 [1.1, 2.0]	2.0 [1.5, 2.4]	<0.001
LVEF, mean (SD), %	57.2 (10.0)	61.6 (7.5)	0.002
LVESD, median (IQR), cm	3.0 [2.8, 3.9]	2.9 [2.6, 3.3]	0.01
LVEDD, median (IQR), cm	5.3 [4.8, 5.8]	4.5 [4.2, 4.9]	<0.001
IVS, median (IQR), cm	0.9 [0.8, 1.0]	1.2 [1.0, 1.5]	<0.001
PWT, median (IQR), cm	0.8 [0.8, 1.0]	1.0 [0.8, 1.1]	0.006
LVESV, median (IQR), ml	41.0 [37.5, 66.0]	32.0 [23.0, 40.0]	0.003
LVEDV, median (IQR), ml	141.0 [111.0, 148.5]	88.0 [67.2, 104.8]	<0.001
Ascending aorta diameter, median (IQR), cm	3.2 [3.0, 3.4]	3.6 [3.0, 4.3]	0.003
Sinus of Valsalva, median (IQR), cm	3.2 [2.9, 3.4]	3.3 [2.9, 3.7]	0.33
Sinotubular junction, median (IQR), cm	2.5 [2.3, 2.6]	2.9 [2.6, 3.2]	0.22
Aorta diameter > 4cm, No. (%)	4 (11.4)	85 (34.7)	0.01

Continuous variables are reported using the median value with interquartile range (IQR); categorical variables are presented as numbers and percentages. Abbreviations: BAV, bicuspid aortic valve; QAV, quadricuspid aortic valve; BSA, body surface area; BMI, body mass index; AS, aortic stenosis; AR, aortic regurgitation; MAVD, mixed aortic valve disease; LVEF, left ventricular ejection fraction; LVESD,

© 2025 Zhang J et al. *JAMA Network Open*.

left ventricular end-systolic diameter; LVEDD, left ventricular end-diastolic diameter; IVS, interventricular sepal diameter; PWT, posterior wall thickness; LVESV, left ventricular end-systolic volume; LVEDV, left ventricular end-diastolic volume; AV, aortic valve.

© 2025 Zhang J et al. *JAMA Network Open*.

eTable 5. Baseline characteristics in patients with valvular dysfunction

Characteristic	QAV N=104	BAV N=450	*P* value
**Demographics**			
Age, median (IQR), years	53.8 [47.7, 62.9]	57.1 [47.6, 65.4]	0.23
Male, No. (%)	65 (62.5)	283 (62.9)	>.99
Height, median (IQR), cm	168.0 [160.8, 172.0]	164.0 [158.0, 171.0]	0.004
Weight, median (IQR), kg	67.8 [60.0, 75.0]	66.0 [56.0, 74.2]	0.06
BSA, median (IQR), m^2	1.8 [1.6, 1.9]	1.7 [1.6, 1.9]	0.02
BMI, median (IQR), kg/m^2	24.4 [22.3, 26.0]	24.0 [21.9, 26.5]	0.62
**Comorbidities**			
Hypertension, No. (%)	57 (54.8)	169 (37.6)	0.002
Hyperlipidemia, No. (%)	44 (42.3)	102 (22.7)	<0.001
Diabetes mellitus, No. (%)	12 (11.5)	57 (12.7)	0.88
Coronary artery disease, No. (%)	17 (16.3)	112 (24.9)	0.08
Atrial fibrillation, No. (%)	5 (4.8)	19 (4.2)	>.99
Prior or baseline heart failure, No. (%)	13 (12.5)	40 (8.9)	0.34
**Echocardiography**			
Valve function, No. (%)			<0.001
Isolated AS	1 (1.0)	261 (58.0)	
Isolated AR	99 (95.2)	133 (29.6)	
MAVD	4 (3.8)	56 (12.4)	
AV peak gradient, median (IQR), mmHg	16.0 [10.2, 25.5]	57.9 [31.1, 84.9]	<0.001
AV mean gradient, median (IQR), mmHg	7.6 [5.1, 12.5]	35.0 [17.0, 51.0]	<0.001
AV peak velocity, median (IQR), m/s	2.0 [1.6, 2.6]	3.8 [2.8, 4.7]	<0.001
LVEF, mean (SD), %	60.0 [52.0, 65.0]	60.0 [55.0, 65.0]	0.03
LVESD, median (IQR), cm	4.0 [3.5, 5.3]	3.2 [2.7, 4.0]	<0.001
LVEDD, median (IQR), cm	6.0 [5.5, 6.7]	4.9 [4.3, 5.7]	<0.001
LVESV, median (IQR), ml	66.0 [51.0, 124.0]	43.0 [26.4, 65.2]	<0.001
LVEDV, median (IQR), ml	167.0 [147.0, 255.0]	108.0 [77.0, 154.0]	<0.001
IVS, median (IQR), cm	1.0 [0.9, 1.2]	1.5 [1.2, 1.7]	<0.001
PWT, median (IQR), cm	1.0 [0.8, 1.1]	1.2 [1.0, 1.3]	<0.001
Ascending aorta diameter, median (IQR), cm	3.6 [3.4, 4.1]	4.0 [3.4, 4.5]	0.002
Sinus of Valsalva, median (IQR), cm	3.4 [3.2, 3.9]	3.3 [3.0, 3.8]	0.19
Sinotubular junction, median (IQR), cm	2.8 [2.8, 3.1]	3.1 [2.6, 3.5]	0.81
Aorta diameter >4 cm, No. (%)	39 (37.5)	236 (52.4)	0.008

Abbreviations refer to eTable 4.

© 2025 Zhang J et al. *JAMA Network Open*.

eTable 6. Baseline characteristics of patients who underwent cardiac computed tomography scanning

Characteristic	BAV<br>N=250	QAV<br>N=107	*P* value
Interval from baseline TTE, mean (SD), days	28.5±72.0	31.2±87.2	0.76
**Demographics**			
Age, median (IQR), years	56.4 [50.0, 63.7]	53.8 [47.6, 63.3]	0.20
Male (%)	153 (61.2)	70 (65.4)	0.52
Height, median (IQR), cm	166.0 [160.0, 172.0]	168.0 [160.5, 172.0]	0.10
Weight, median (IQR), kg	68.3 [60.0, 75.0]	68.0 [61.0, 77.0]	0.44
BSA, m^2	1.8 [1.6, 1.9]	1.8 [1.7, 1.9]	0.27
BMI, kg/m^2	24.5 [22.3, 26.7]	24.5 [22.2, 26.5]	0.86
**Comorbidities**			
Hypertension (%)	122 (48.8)	60 (56.1)	0.25
Hyperlipidemia (%)	90 (36.0)	41 (38.3)	0.76
Diabetes mellitus (%)	33 (13.2)	13 (12.1)	0.92
Coronary artery disease (%)	66 (26.4)	16 (15.0)	0.02
Atrial fibrillation (%)	8 (3.2)	6 (5.6)	0.43
Prior or baseline heart failure (%)	29 (11.6)	11 (10.3)	0.85
**Echocardiography**			
Valve Function (%)			
No significant valvular dysfunction	78 (31.2)	21 (19.6)	<0.001
Isolated AS	108 (43.2)	1 (0.9)	
Isolated AR	43 (17.2)	81 (75.7)	
MAVD	21 (8.4)	4 (3.7)	
AV peak gradient, median (IQR), mmHg	43.6 [21.0, 82.5]	14.4 [10.2, 24.0]	<0.001
AV mean gradient, median (IQR), mmHg	23.5 [11.0, 50.0]	6.5 [4.5, 10.6]	<0.001
AV peak velocity, median (IQR), m/s	3.3 [2.3, 4.6]	1.9 [1.6, 2.5]	<0.001
LVEF, mean (SD), %	61.0 (10.2)	56.7 (10.5)	<0.001
LVESD, median (IQR), cm	3.2 [2.8, 3.8]	4.0 [3.4, 5.2]	<0.001
LVEDD, median (IQR), cm	4.9 [4.5, 5.6]	5.8 [5.4, 6.6]	<0.001
LVESV, median (IQR), ml	41.0 [27.0, 62.0]	66.0 [49.0, 113.0]	<0.001
LVEDV, median (IQR), ml	108.0 [88.0, 157.2]	163.5 [145.5, 249.0]	<0.001
IVS, median (IQR), cm	1.2 [1.0, 1.4]	1.0 [0.9, 1.1]	<0.001
PWT, median (IQR), cm	1.0 [0.9, 1.2]	0.9 [0.8, 1.1]	<0.001
Ascending aorta diameter, median (IQR), cm	4.3 [3.7, 4.8]	3.6 [3.3, 4.0]	<0.001
Sinus of Valsalva, median (IQR), cm	3.5 [3.1, 3.9]	3.3 [3.2, 3.6]	0.80
Sinotubular junction, median (IQR), cm	3.2 [2.9, 3.5]	3.1 [2.3, 3.2]	0.78
Aorta diameter >4 cm, (%)	168 (67.2)	35 (32.7)	<0.001

TTE, transthoracic echocardiography; abbreviations refer to eTable 4.

© 2025 Zhang J et al. *JAMA Network Open*.

eTable 7. Baseline characteristics of patients who underwent cardiac magnetic resonance scanning

Characteristic	QAV N=21	BAV N=35	*P* value
Interval from baseline TTE, mean (SD), days	214.0±138.9	234.4±133.7	0.56
**Demographics**			
Age, median (IQR), years	53.8 [42.9, 62.0]	55.2 [36.4, 60.6]	0.64
Male, No. (%)	13 (61.9)	20 (57.1)	0.94
Height, median (IQR), cm	168.0 [165.0, 172.0]	165.0 [160.0, 173.0]	0.53
Weight, median (IQR), kg	64.0 [60.0, 71.0]	69.0 [57.5, 75.0]	0.94
BSA, median (IQR), m^2	1.7 [1.6, 1.8]	1.8 [1.6, 1.8]	0.99
BMI, median (IQR), kg/m^2	23.5 [21.8, 24.2]	23.2 [21.5, 26.1]	0.89
**Comorbidities**			
Hypertension, No. (%)	11 (52.4)	13 (37.1)	0.40
Hyperlipidemia, No. (%)	10 (47.6)	7 (20.0)	0.06
Diabetes mellitus, No. (%)	2 (9.5)	5 (14.3)	0.91
Coronary artery disease, No. (%)	7 (33.3)	7 (20.0)	0.42
Atrial fibrillation, No. (%)	3 (14.3)	3 (8.6)	0.82
Prior or baseline heart failure, No. (%)	10 (47.6)	8 (22.9)	0.10
**Echocardiography**			
Valve Function, No. (%)			
No significant valvular dysfunction	7 (33.3)	19 (54.3)	0.001
Isolated AS	0 (0.0)	10 (28.6)	
Isolated AR	13 (61.9)	6 (17.1)	
MAVD	1 (4.8)	0 (0.0)	
AV peak gradient, median (IQR), mmHg	13.0 [9.0, 21.2]	23.0 [14.0, 44.2]	0.01
AV mean gradient, median (IQR), mmHg	5.8 [4.5, 10.6]	12.0 [7.5, 26.0]	0.002
AV peak velocity, median (IQR), m/s	1.8 [1.5, 2.3]	2.3 [2.0, 3.1]	0.01
LVEF, mean (SD), %	45.0 (13.1)	55.2 (13.4)	0.008
LVESD, median (IQR), cm	5.1 [3.7, 6.1]	3.2 [2.9, 4.0]	0.01
LVEDD, median (IQR), cm	6.7 [5.8, 7.1]	4.8 [4.4, 5.6]	<0.001
LVESV, median (IQR), ml	88.0 [66.0, 247.0]	36.5 [25.2, 78.8]	0.05
LVEDV, median (IQR), ml	220.0 [153.5, 267.0]	105.0 [80.2, 147.8]	0.006
IVS, median (IQR), cm	1.0 [0.9, 1.2]	1.3 [1.0, 1.6]	0.02
PWT, median (IQR), cm	0.9 [0.8, 1.1]	1.0 [0.9, 1.2]	0.13
Ascending aorta diameter, median (IQR), cm	3.4 [3.1, 4.0]	3.9 [3.3, 4.5]	0.12
Sinus of Valsalva, median (IQR), cm	3.3 [3.2, 3.5]	3.5 [2.8, 3.8]	0.89
Sinotubular junction, median (IQR), cm	2.7 [2.4, 2.9]	2.9 [2.5, 3.0]	0.86
Aorta diameter >4 cm, No. (%)	6 (28.6)	16 (45.7)	0.32

Abbreviations refer to eTable 4.

© 2025 Zhang J et al. *JAMA Network Open*.

eTable 8. Characteristics of eight patients with coexisting cardiomyopathy and quadricuspid aortic valves

Characteristic	Patient #1	Patient #2	Patient #3	Patient #4	Patient #5	Patient #6	Patient #7	Patient #8
**Demographics and Comorbidities**								
Age, years	36	51	68	56	42	47	72	57
Gender	Male	Female	Female	Male	Male	Female	Male	Male
Hypertension	yes	no	no	no	yes	no	yes	yes
Hyperlipidemia	yes	no	yes	no	no	no	no	no
Diabetes mellitus	no	no	no	no	no	no	no	no
Coronary artery disease	no	no	no	no	no	no	no	yes
Atrial fibrillation	no	no	no	no	no	no	no	no
Prior or baseline heart failure	yes	no	yes	yes	yes	no	no	no
Ventricular arrythmia	yes	no	yes	no	no	no	no	no
**Echocardiographic findings**								
AR severity	Severe	Moderate-to-severe	Mild	Moderate	Mild	Moderate-to-severe	Mild	Trivial
LVEF, %	47	56	43	28	40	46	45	70

Continued

© 2025 Zhang J et al. *JAMA Network Open*.

Continued								
	Patient #1	Patient #2	Patient #3	Patient #4	Patient #5	Patient #6	Patient #7	Patient #8
Aorta diameter > 4cm	yes	yes	no	no	no	no	no	yes
**CMR findings**								
Cardiomyopathy type	LVNC	HCM+LVNC	LVNC	HCM	DCM	LVNC	DCM	HCM
Diagnosis description	LV trabeculations in apical and inferior segments with deep intertrabecular recesses with noncompacted: compacted LV wall ratio 2:1	Nonobstructive HCM. IVS diameter: 2.8cm. LV trabeculations in apical segment with noncompacted: compacted LV wall ratio 2:1	Enlarged LV dimensions with reduced systolic function (LVEF 51%). Apical segment noncompacted: compacted LV wall ratio 2:1.	Globally LV hypertrophy (wall thickness 2.0cm). LV trabeculations in apical segment with noncompacted: compacted LV wall ratio 1.5:1	Enlarged LV dimensions with reduced systolic function (LVEF 21.3%).	LV trabeculations with deep intertrabecular recesses. Noncompacted: compacted LV wall ratio > 2:1	Enlarged LV dimensions with reduced systolic function (LVEF 44%).	Nonobstructive HCM. Globally LV hypertrophy (wall thickness 1.8cm). No aortic valve stenosis.
LGE present	no	yes	no	no	yes	no	no	no
AR severity	Moderate-to-severe	Severe	Mild	Moderate-to-severe	Trivial	Moderate-to-severe	Mild	Trivial
LVEF, %	40.9	52	47	31	21.3	37.1	44	66
LVESV, ml	116	80.8	84	354	284	143	118	44
LVEDV, ml	197	174.6	172	367	361	228	224	141

© 2025 Zhang J et al. *JAMA Network Open*.

LV noncompaction	yes	yes	yes	no	no	yes	no	no

CMR, cardiac magnetic resonance; LGE, late gadolinium enhancement; LVNC, left ventricular noncompaction; HCM, hypertrophic cardiomyopathy; DCM, dilated cardiomyopathy. Other abbreviations refer to eTable 4.

© 2025 Zhang J et al. *JAMA Network Open*.

eTable 9. Factors associated with heart failure hospitalization in patients with quadricuspid and bicuspid aortic valves from multivariable regression analysis *

Characteristic	Patients with QAV		Patients with BAV	
	HR (95% CI)	*P* Value	HR (95% CI)	*P* Value
Age (per year)	1.01 (0.99, 1.04)	0.26	1.02 (1.01, 1.04)	0.003
Male	2.37 (1.08, 5.20)	0.03	1.99 (1.34, 2.96)	<0.001
Hypertension	0.86 (0.43, 1.70)	0.65	0.83 (0.51, 1.32)	0.42
Hyperlipidemia	0.74 (0.36, 1.52)	0.41	0.91 (0.57, 1.43)	0.67
Diabetes mellitus	0.83 (0.23, 3.01)	0.78	1.77 (1.07, 2.93)	0.02
Coronary artery disease	1.70 (0.73, 3.95)	0.21	1.17 (0.74, 1.84)	0.49
Atrial fibrillation	3.05 (1.08, 8.61)	0.03	2.56 (1.29, 5.08)	0.007
Presence of cardiomyopathies	4.27 (2.26, 8.07)	<0.001	1.73 (0.26, 11.4)	0.57
Severe AS	/	/	2.26 (1.33, 3.85)	0.003
Severe AR	2.63 (1.04, 6.64)	0.04	3.12 (1.94, 5.01)	<0.001

* Variables associated with HFH in patients with BAV and QAV were assessed separately in Fine-Gray regression models in which all-cause death was considered as the competing event. HFH, heart failure hospitalization. Other abbreviations refer to eTable 4.

© 2025 Zhang J et al. *JAMA Network Open*.

eFigure 1. Study flowchart

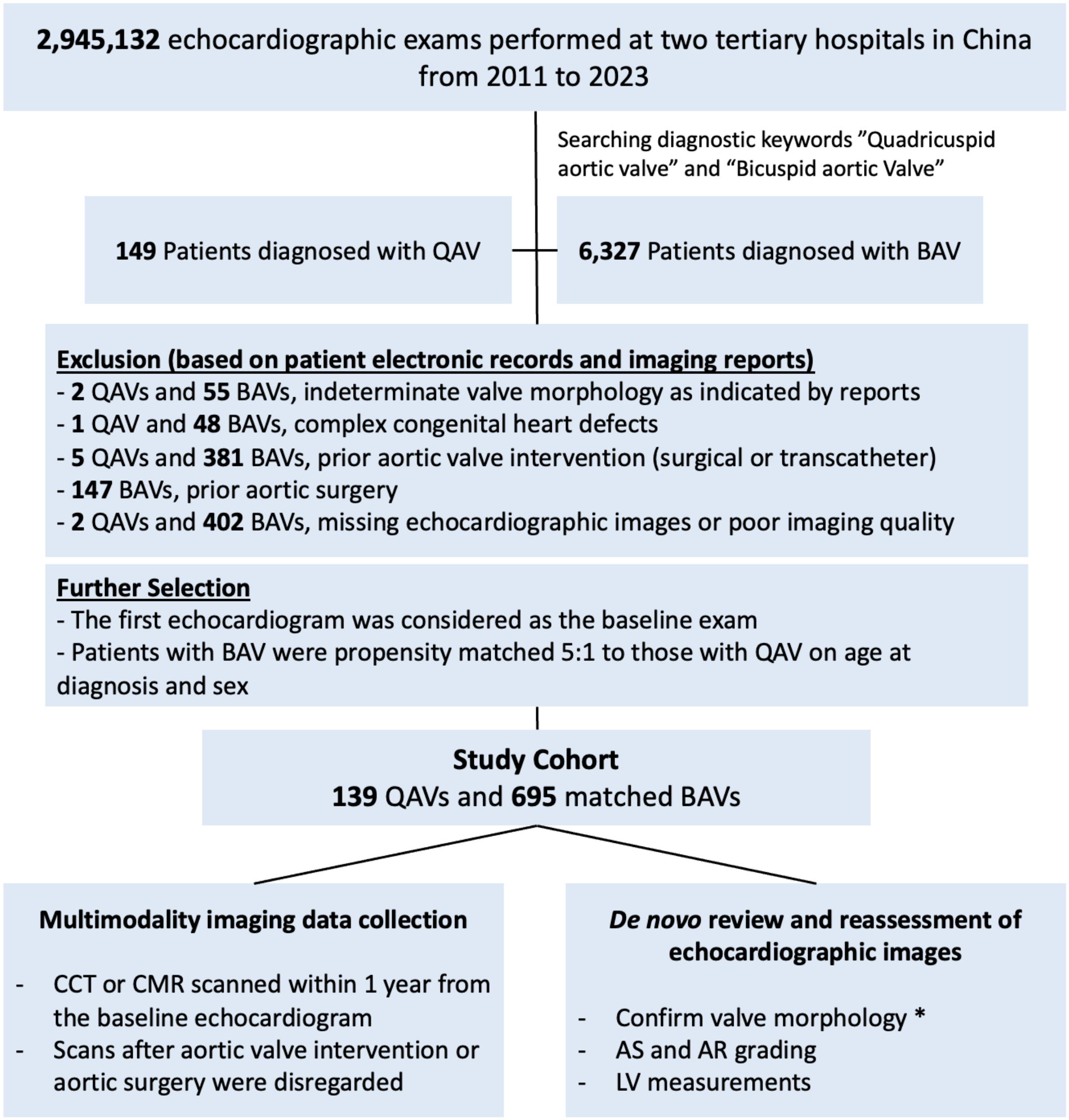

Legends: * During reviewing, no QAV cases were excluded, while two BAV cases were removed from the cohort (one tricuspid aortic valve, one indeterminate valve morphology). Subsequently, two random BAV cases of the same age and sex were selected as replacements. QAV, quadricuspid aortic valve; BAV, bicuspid aortic valve; AS, aortic stenosis; AR, aortic regurgitation. CCT, cardiac computed tomography; CMR, cardiac magnetic resonance.

© 2025 Zhang J et al. *JAMA Network Open*.

eFigure 2. Morphologic assessment of aortic valve using echocardiography

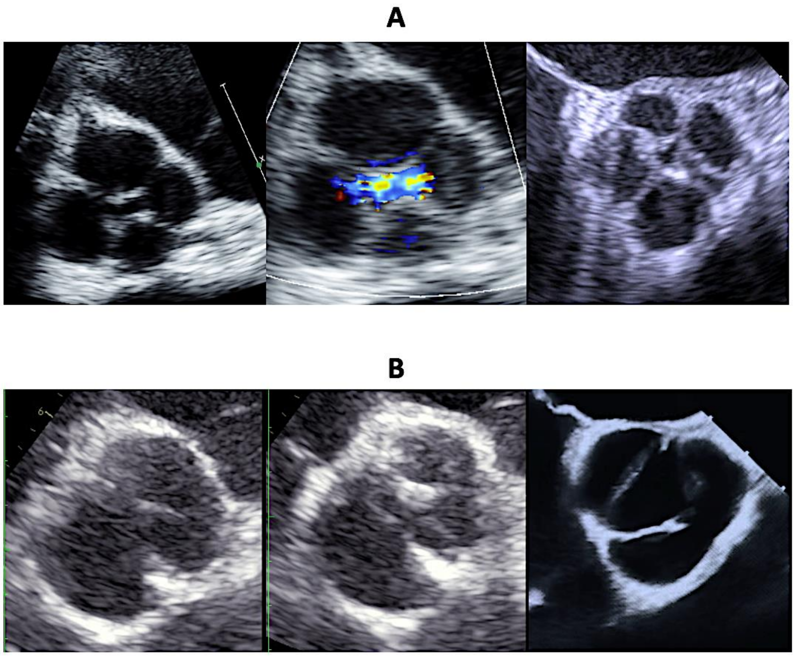

Legends: Parasternal short-axis views at aortic valve level were reviewed to confirm the valve morphology. QAV was identified by the presence of four cusps and inter-leaflet commissures, with or without a central orifice during diastole (**Panel A**). BAV was characterized by an aortic valve with a partial or complete obliteration of the commissure between two adjacent cusps, with or without a raphe, resulting in a “fish mouth”-like systolic opening of the valve (**Panel B**).

eFigure 3. Proportion of missing values for the main study variables

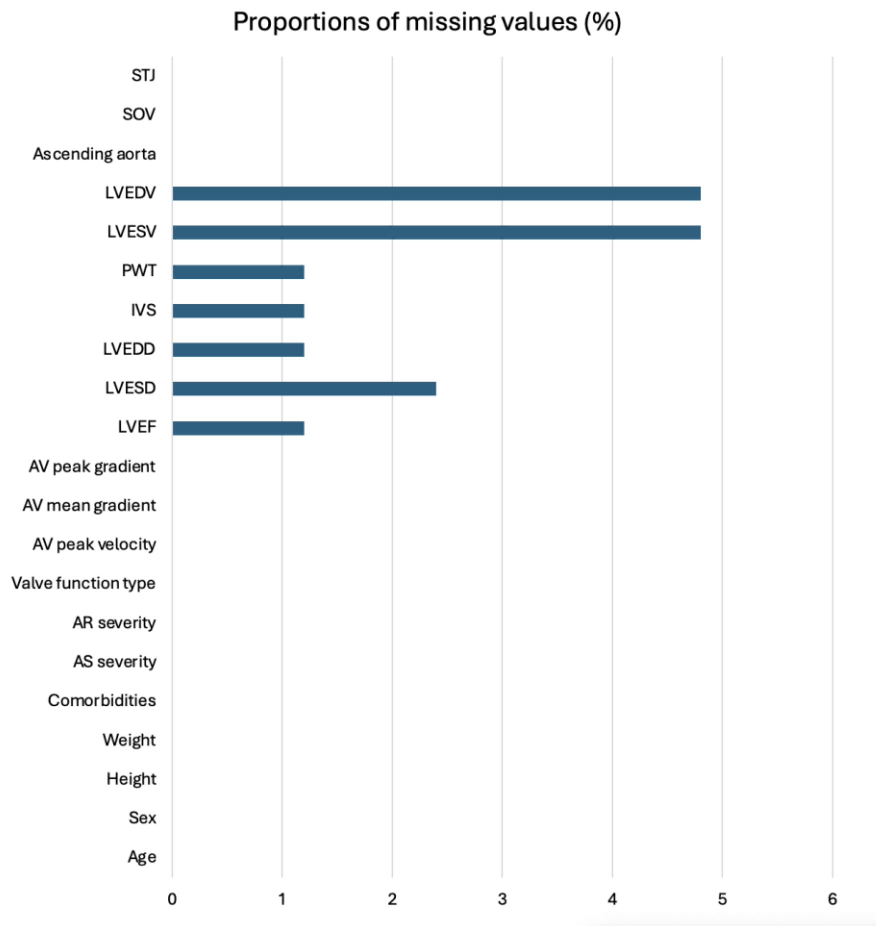

Legends: BSA, body surface area; BMI, body mass index; AS, aortic stenosis; AR, aortic regurgitation; MAVD, mixed aortic valve disease; LVEF, left ventricular ejection fraction; LVESD, left ventricular end-systolic diameter; LVEDD, left ventricular end-diastolic diameter; IVS, interventricular sepal diameter; PWT, posterior wall thickness; LVESV, left ventricular end-systolic volume; LVEDV, left ventricular end-diastolic volume; AV, aortic valve.

© 2025 Zhang J et al. *JAMA Network Open*.

eFigure 4. Aortic valve calcium score assessed by computed tomography scanning

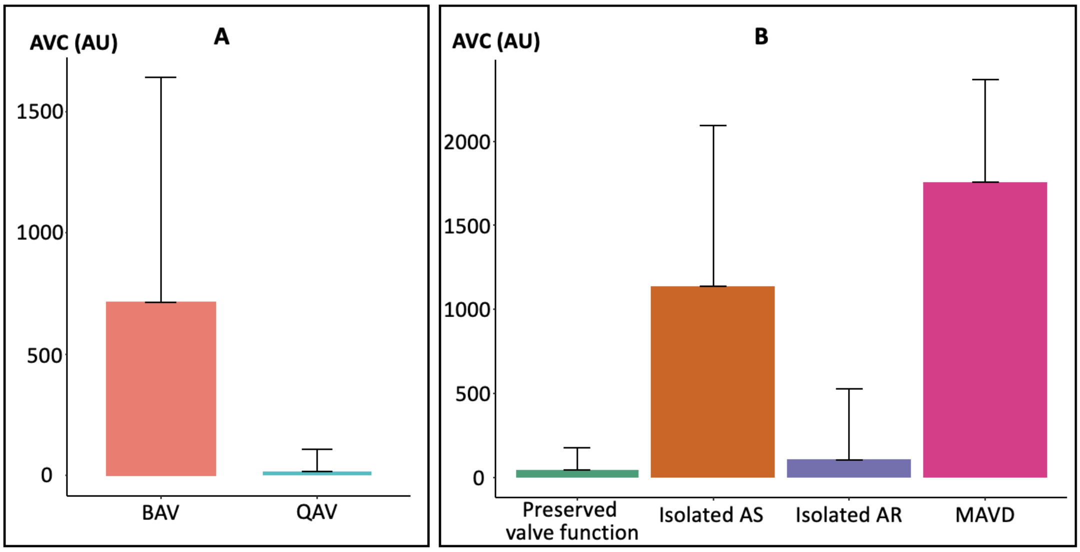

Legends: **Panel A** shows the mean AVC score between patients with BAV and QAV. **Panel B** displays the mean AVC score of BAV group categorized by baseline valve function. AVC, aortic valve calcium; AU, Agatston units; AS, aortic stenosis; AR, aortic regurgitation; MAVD, mixed aortic valve disease.

© 2025 Zhang J et al. *JAMA Network Open*.

eFigure 5. Coronary artery anomalies in patients with quadricuspid and bicuspid aortic valves

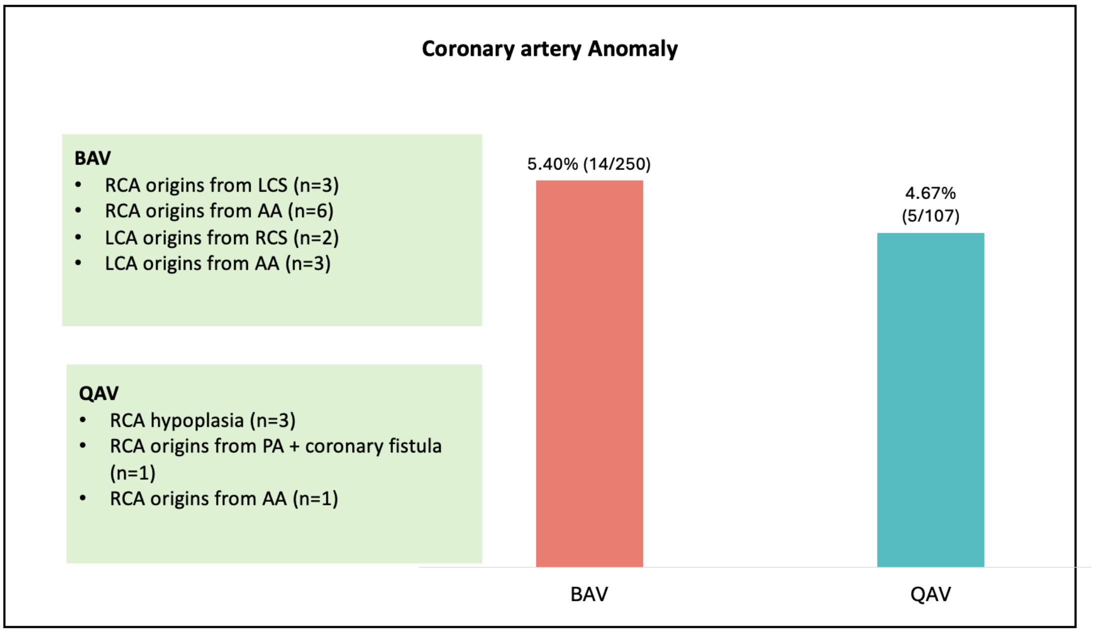

Legends: RCA, right coronary artery; LCA, left coronary artery; LCS, left coronary sinus; RCS, right coronary sinus; AA, ascending aorta; PA, pulmonary artery.

© 2025 Zhang J et al. *JAMA Network Open*.
