# Supplementary material for: Clinical Outcomes in Patients With Quadricuspid vs Bicuspid Aortic Valve
Source: JAMA Netw Open. 2025 Aug 4;8(8):e2524915. doi: 10.1001/jamanetworkopen.2025.24915 (PMC12322796; doi:10.1001/jamanetworkopen.2025.24915)
Supplement: Supplement 2. — Data Sharing Statement [file jamanetwopen-e2524915-s002.pdf]

Data Sharing Statement

Zhang. Clinical Outcomes in Patients With Quadricuspid vs Bicuspid Aortic Valve. *JAMA Netw Open*. Published August 04, 2025. doi:10.1001/jamanetworkopen.2025.24915

Data
Data available: No

Additional Information
Explanation for why data not available: The data underlying this article will be shared on reasonable request to the corresponding authors.
